# Mobility networks in Greater Mexico City

**DOI:** 10.1038/s41597-023-02880-y

**Published:** 2024-01-18

**Authors:** Marisol Flores-Garrido, Guillermo de Anda-Jáuregui, Plinio Guzmán, Amilcar Meneses-Viveros, Alfredo Hernández-Álvarez, Erika Cruz-Bonilla, Maribel Hernández-Rosales

**Affiliations:** 1 Escuela Nacional de Estudios Superiores Unidad Morelia, Universidad Nacional Autónoma de México, Antigua Carretera a Pátzcuaro 8701, Indeco la Huerta, Ciudad de México, 58190 Michoacan Mexico; 2 National Institute of Genomics Medicine, Periferico Sur 4809, Arenal Tepepan, Tlalpan, 14610 Mexico City, Mexico; 3 National Council for Science and Technology, Av. Insurgentes Sur 1582, Colonia Crédito Constructor, Benito Juárez, Mexico City, Mexico; 4 Estornuda.me, Mexico City, Mexico; 5 Center for Research and Advanced Studies of IPN, Av Instituto Politécnico Nacional 2508, San Pedro Zacatenco, Gustavo A. Madero, 07360 Mexico City, Mexico; 6 Center for Genomics Sciences, Universidad Nacional Autónoma de México, Avenida Universidad s/n, Universidad Autonoma del Estado de Morelos, 62210 Cuernavaca, Morelos Mexico; 7 Center for Research and Advanced Studies of IPN, Irapuato Unit, Libramiento Norte Carretera Irapuato León Kilómetro 9.6, 36821 Irapuato, Guanajuato Mexico

**Keywords:** Scientific data, Computer science

## Abstract

Based on more than 11 billion geolocated cell phone records from 33 million different devices, daily mobility networks were constructed over a 15-month period for Greater Mexico City, one of the largest and most diverse metropolitan areas globally. The time frame considered spans the entire year of 2020 and the first three months of 2021, enabling the analysis of population movement dynamics before, during, and after the COVID-19 health contingency. The nodes within the 456 networks represent the basic statistical geographic areas (AGEBs) established by the National Institute of Statistics, Geography, and Informatics (INEGI) in Mexico. This framework facilitates the integration of mobility data with numerous indicators provided by INEGI. Edges connecting these nodes represent movement between AGEBs, with edge weights indicating the volume of trips from one AGEB to another. This extensive dataset allows researchers to uncover travel patterns, cross-reference data with socio-economic indicators, and conduct segregation studies, among other potential analyses.

## Background & Summary

Today’s cities and the lifestyles they offer are greatly influenced by mobility. Employing complex networks to depict a city’s mobility has emerged as a powerful method for modeling population dynamics across various scales and discerning collective travel trends^1,2^. The daily commutes of urban residents are intricately linked to the city’s attributes, including its transportation network, established commercial hubs, areas hosting schools, workplaces, recreational facilities, and medical services, among others. Mobility networks are pivotal in visualizing interactions among various urban areas and establishing spatial patterns. They offer significant utility for decision-making, strategizing for risk scenarios, detecting vulnerabilities, and gaining deeper insights into the unique characteristics of localities within urban landscapes.

Furthermore, these urban mobility networks have a broad spectrum of applications, encompassing the examination of mobility behavior^3–6^, optimization of traffic flow^7,8^, management of parking demand^9,10^, promotion of sustainable transport^11,12^, evaluation of environmental impact^13,14^, event planning^15–21^, infrastructure development^22,23^, and public transportation planning^24–30^. These networks contribute to understanding mobility patterns, traffic optimization, sustainability promotion, and environmental impact analysis, and they inform event management, infrastructure planning, and optimization of public transportation systems. Moreover, mobility networks find applications in socio-economic studies, including investigations related to pandemic-related mobility^31–33^ and migration analysis^34^.

Constructing mobility networks requires records of the location of a group of people over time, so that their travel can be estimated and routes can be established that, when aggregated, describe patterns of population movement. There are studies that use social network data to infer the movements of people in a given area, for example, geolocated Twitter information^35–37^. This has the disadvantage of having data biased towards sectors of the population that use certain platforms and specific apps. Among the data sources for building mobility networks, cell phone records represent the alternative that guarantees the best sampling; cell phone use covers a wide range of population characteristics, especially in urban areas. However, these data are not easy to obtain: they involve costs associated with managing the records with private companies and processing them to achieve a format that allows the analysis of the information.

Greater Mexico City, with approximately 22 million inhabitants, is among the largest metropolitan areas in the world. However, in contrast to the availability of data on other large cities, we could locate only one work that analyzes mobility patterns in Mexico with a network approach based on Twitter data^38^.

**Fig. 1:**
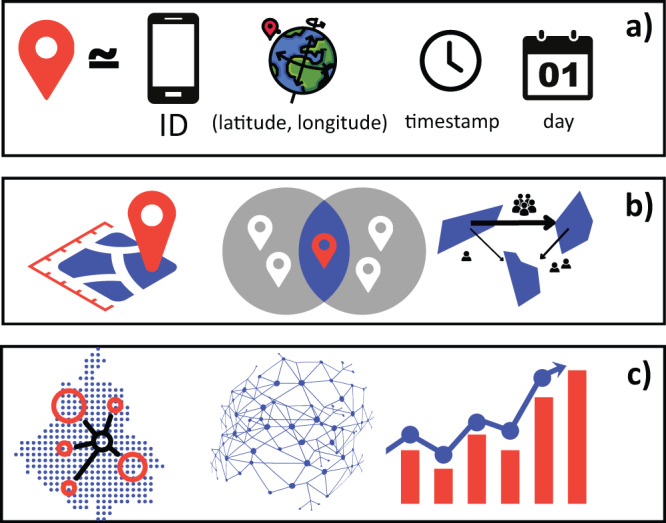
Schematic overview of the study. (**a**) Input data: anonymized device IDs, locations, and timestamps. (**b**) Device assignment to AGEBs: we geolocate each device to the AGEB where it is observed during the day, identify device counts in all AGEB pairs, and assign them as edge weights in the mobility network. (**c**) Mobility networks: nodes representing regions in Greater Mexico City enable the study of network properties over time.

In this work we managed to obtain and process an average of 25 million records per day, during a period of fifteen months, corresponding to cell phone-based location data of inhabitants of Greater Mexico City. Then, we aggregated the information to build a set of 456 mobility networks between zones in Greater Mexico City. To establish the zones we were guided by the basic geostatistical areas (AGEBs) defined by the National Institute of Statistics, Geography and Informatics^39^. Each of these areas corresponds to a census block group and can be easily linked to studies that determine its socioeconomic profile. Figure 1 presents a schematic overview detailing the methodology employed in this study.

The dataset represents an important contribution due to the amount of data processed and the work invested in structuring them in mobility networks, following a reconstruction based on mobile device location data or *pings*. In addition, the data (1) is organized with a time axis that allows the study of mobility over fifteen months, and (2) covers the year 2020, so that it is possible to analyze the disruption in the behavior of the population derived from the health contingency of SARS-Cov-2.

In fact, the recent COVID-19 crisis has underscored the significance of human flow datasets across diverse geographical scales. These datasets support evidence-based public health policies and various applications related to social sensing and transportation. To illustrate, the work by Guidotti *et al*.^40^ presents time-series data encompassing various parameters associated with the health crisis arising from Covid-19 in 230 countries. Similar datasets explore specific regions; for example, Liu *et al*.^41^ provide a detailed account of cases in China, integrating demographic, clinical, and mobility information. This intersection of Covid-19 cases and mobility within a region is also evident in datasets released by Ponce de Leon *et al*.^42^ for Spain, Pepe *et al*.^43^ for Italy, and Kang *et al*.^44^ for the United States. These research groups aggregate information and present it through mobility networks, spatial networks, or descriptive sets of edges, respectively, recognizing the importance of studying the unique characteristics of each country, including territorial organization, population size, and public policies. As highlighted by Kang *et al*.^44^, examining human flow at various geographic scales enhances our understanding of human dynamics during a health crisis.

Mexico represents one of the countries that did not have a strict lockdown. With an economy heavily based on services and informal trade, the country faced the crisis with a dilemma between emergency measures to manage the health sector crisis and keep the economy functioning. Far from strict measures, the government implemented different recommendations that had a very dissimilar effect among different sectors of the population, mainly between those who could work from home and isolate themselves and those who were forced to provide services to keep the city running. The difference in the situation of different sectors of the population translates into different patterns of displacement in AGEBS, depending, in part, on the economic characteristics of the areas.

Crisis management itself makes the study of mobility patterns in different countries a completely local issue. We believe that the dataset made available to the community will allow a deeper understanding of the particular dynamics of a large city in Latin America and the specific ways in which a crisis breaks patterns and creates a new, and differentiated, everyday life.

## Methods

For this research study, we procured data from Veraset, a data-as-a-service company that offers a range of datasets, including cell phone location data^45^. The data provided by this company are subjected to anonymization procedures and are presented in a tabular format. Each data point (ping) allows us to ascertain an anonymous user’s geographical coordinates (latitude and longitude) at a specific timestamp.

**Fig. 2:**
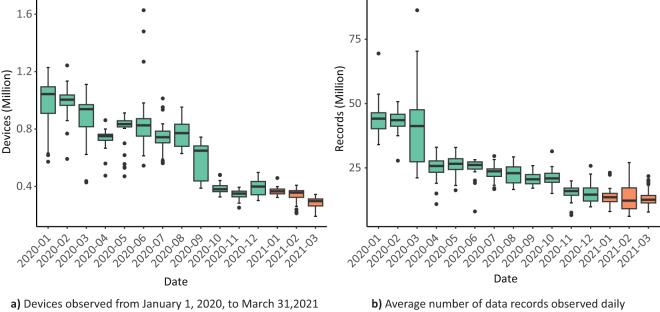
Devices (**a**) and data point records (**b**) processed per month to build the mobility networks. Data for 2020 and 2021 is shown in green and orange, respectively.

The temporal scope of the dataset used in our study spans from January 1, 2020, to March 31, 2021, and pertains exclusively to geographic records within the borders of Mexico. The dataset for Greater Mexico City comprises a total of 11 billion records from 33 million devices, with approximately 0.4 to 1.2 million devices and records ranging from 10 to 50 million per day, as illustrated in Fig. 2. These data points’ temporal resolution extended to the level of seconds. There is a confidentiality agreement with Veraset specifying that publications are restricted to presenting results derived solely from aggregated information or estimated model parameters. Nonetheless, in the interest of transparency and reproducibility, we have made available an illustrative example of the source dataset on the Open Science Framework (OSF)*.

Using data from mobile phone usage holds significant promise for facilitating an accurate estimation of population mobility. Notably, Mexico City, the capital of Mexico situated within the broader metropolitan region known as Greater Mexico City, stands out as the federal entity with the highest cellular telephony adoption. As of 2021, approximately 88.4% of its population was reported to have access to this technology^46^.

In order to build the mobility networks, it is necessary to locate cell phone devices to previously established geographic units. In the construction of this dataset we use the basic geostatistical areas (AGEBs) defined by the National Institute of Statistics, Geography and Informatics (INEGI), as nodes of the networks. AGEBs are the fundamental unit of the Institute’s geostatistical framework and are adjusted to the state and municipal boundaries of the political-administrative division of the country.

**Fig. 3:**
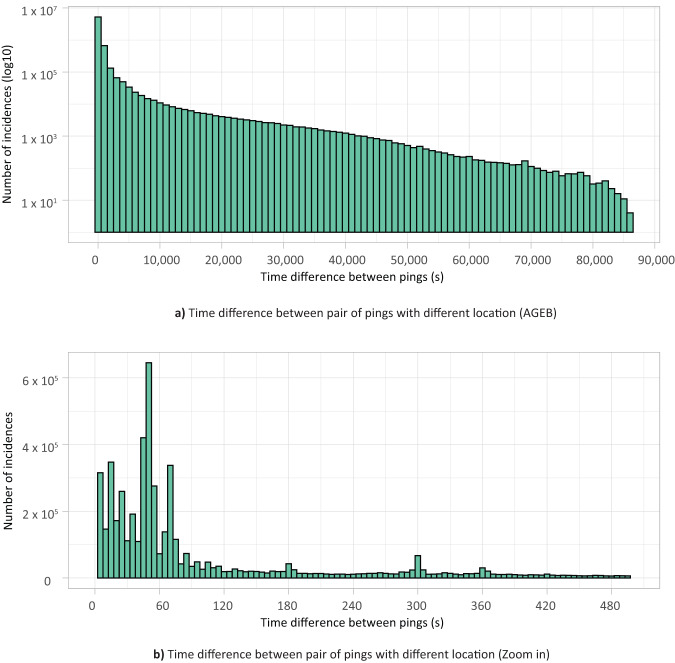
Distribution of the time difference found between pair of records where a change in location was detected. Most of the crossover between AGEBs occur between pair of records with a time difference below 120 seconds.

Therefore, we aggregate devices into the respective AGEBs where they are observed to create study units. The aggregation process commences by identifying all AGEBs where a device has been recorded, with this identification being performed daily for each device. Subsequently, we aggregate all devices observed at origin nodes and similarly at destination nodes. As a result, our reporting centers on the count of devices observed transitioning from one AGEB to another, represented by edge weights. Furthermore, we define travel time as the period during which a device moves from one AGEB to another between two consecutive time points. Upon analyzing the time taken by each device for this journey, we have determined that, in most cases, it falls within the range of 1 to 120 seconds, as illustrated in Fig. 3. Therefore, we consider all travels lasting at least one second.

The area considered in this study is delimited according to INEGI’s definition of the Metropolitan Zone of the Valley of Mexico, which covers about 7,855 km^2^ and includes the 16 municipalities of Mexico City, 59 municipalities of the state of Mexico, and one municipality of the state of Hidalgo. Regarding our study units, the area of Greater Mexico City is organized into 5,740 urban AGEBs and 169 rural AGEBs^39^.

**Fig. 4:**
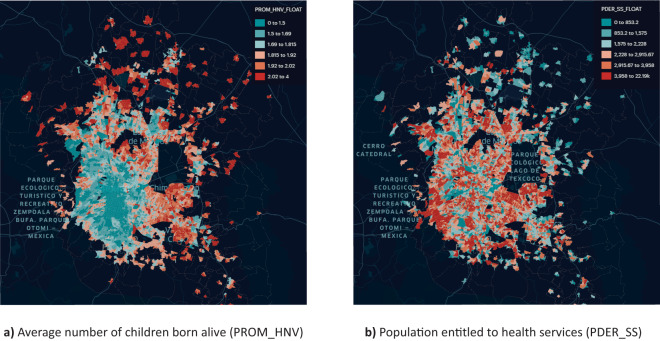
Urban basic geographic units (AGEBs) considered in our analysis of Greater Mexico City correspond to census block groups. The intensity of color in each region is linked to an indicator from the Population and Housing Census conducted by the National Institute of Statistics, Geography, and Informatics (INEGI) in Mexico^47^. The example illustrates the convenience of building the networks following regions defined by INEGI, as this enriches the information on each zone and takes advantage of public access instruments designed by the Institute.

Using AGEBs allows the efficient cross-referencing of information on the areas, for example, with the Institute’s periodic and diverse instruments such as the Population and Housing Census, the National Survey on the Dynamics of Household Relationships, or the National Survey on the Availability and Use of Information Technologies in Households^46–48^. Figure 4 shows some indicators obtained from these surveys for each urban AGEB considered in the region of our analysis. Another analysis that is feasible is to cross-reference data with the marginalization indices reported by INEGI^49^.

Once the geographic units of study have been established, we define the mobility network for a given day *D*_*k*_ as a directed and weighted graph *G*_*k*_ (*V,E*_*k*_), where:

- *V* represents the set of AGEBs within Greater Mexico City.
- An edge (*i*, *j*) ∈ *E*_*k*_ signify mobility, originating from a source node *i* and terminating at a target node *j* during the day *D*_*k*_.
- Mobility in our network is defined based on observing at least one device moving from *i* to *j*. The weight assigned to each edge indicates the fraction of observed devices that have moved from node *i* to node *j* relative to the total number of observed devices during *D*_*k*_, i.e.,

$${W}_{{k}_{ij}}=\frac{\left|{D}_{{k}_{ij}}\right|}{\left|{D}_{k}\right|}\times 1{0}^{6},$$

where $$| {D}_{{k}_{ij}}| $$ is the number of devices that were observed to move from *i* to *j*, that is, were observed in *i* and their next immediate ping after at least one second was in *j*; and $$| {D}_{k}| $$ is the cardinality of the set of unique device ids observed throughout the day *D*_*k*_.

As shown, the weights employed within each network *G*_*k*_ undergo a normalization process relative to the volume of devices observed during the day *D*_*k*_. The normalization procedure constrains the weights ${W}_{{k}_{ij}}$ to a range between 0 and 1, which enhances their interpretability and facilitates comparisons between nodes and across networks within the collection, i.e., across movement flows corresponding to different days. The multiplication by a factor of 10^6^ is employed to manage numerical values efficiently.

Algorithm 1 was developed to construct a network using daily mobile device location data as its input. As depicted in the algorithm, it accomplishes several key tasks. Firstly, it associates each ping with its respective AGEB. Subsequently, it computes the count of observed trips between different areas. Finally, the algorithm normalizes the data following the previously described method.

The complexity to compute these networks is in *O*(*nm* + *l*^2^), where *n* is the number of pings or records processed, *m* is the number of AGEBs and *l* is the number of devices. The quantity of records and devices accessible for network construction varies from day to day. The distribution of records and devices available for network construction is depicted in Fig. 2, with mean values displayed for each month. A minor reduction in the average number of records and devices per day was noted during the early months of 2021. Nevertheless, the normalization of edge weights in the networks enables meaningful comparisons among different days.

### Algorithm 1

Mobility network construction.

## Data Records

The observation period considered in the data covers 456 days, starting January 1st, 2020. Thus, our data consists of 456 mobility networks, each represented by an individual comma-separated values (csv) text file. Each file has an index corresponding to the chronological order of the data; day 1 corresponds to January 1st, 2020, whereas day 456 corresponds to March 31st, 2021. The dataset can be found in the OSF repository: 10.17605/OSF.IO/GWQ6U^50^.

Each node in the network represents an AGEB, defined in accordance with the National Institute of Statistics, Geography and Informatics (INEGI). Each network file contains an edge list of the corresponding network, using three columns. The first two columns indicate the origin and destination nodes, respectively. The third column is a integer representing the edge weight, i.e., the number of trips observed during the day between the connected AGEBs. As described above, the weight of the networks has been normalized.

When the number of observed trips between AGEBs is zero for a given day, the network may contain isolated nodes. These nodes are excluded from the network’s edge list, indicating that not all the csv files comprising the dataset have an identical number of rows.

Finally, in the dataset we have included information on each of the geographic zones considered in the networks. The agebs_ZMVM.csv file indicates, for each zone:

- **IDGEO:** A unique key for each AGEB. It is formed by concatenating the key associated with the entity, municipality, locality (in the case of urban AGEBs), and a specific AGEB key. IDGEO corresponds to ‘CVEGEO’, an identifier commonly encountered in Mexican databases.
- **ID_ENT:** The key of the federal entity according to INEGI.
- **ID_MUN:** The key of the municipality to which the AGEB corresponds.
- **ID_LOC:** In the case of urban AGEBs, the key of the locality to which they belong. This data does not exist for rural AGEBs.
- **ID_AGEB:** The key of the AGEB according to INEGI.
- **Entity:** Name of the federal entity to which the AGEB belongs.
- **Municipality:** Name of the municipality to which each AGEB belongs.
- **Type:** If the AGEB is rural or urban.
- **Long:** Longitude of the centroid of the considered AGEB. This allows us to locate each node of our networks spatially.
- **Lat:** Latitude of the centroid of the considered AGEB. This allows us to locate each node of our networks spatially.
- **Pob_MUN:** Total population of the municipality to which the AGEB belongs, according to the National Population and Housing Census carried out in 2020^47^. Since this is a data corresponding to the municipality, and not to particular AGEBs, it is repeated in all the AGEBs that make up a municipality.

## Technical Validation

The code for the creation of the networks was developed within the team following standard peer review practices. Once the dataset was constructed, three different analyses were implemented to explore the validity and possible limitations of the data.

### Data representativity

To validate the representation of our source dataset to the actual population in Greater Mexico City, a comparison to the data reported by AGEB in the 2020 National Population and Housing Census^47^ was conducted. The devices available for a given date were assigned to a home AGEB according to their first record of the day.

**Fig. 5:**
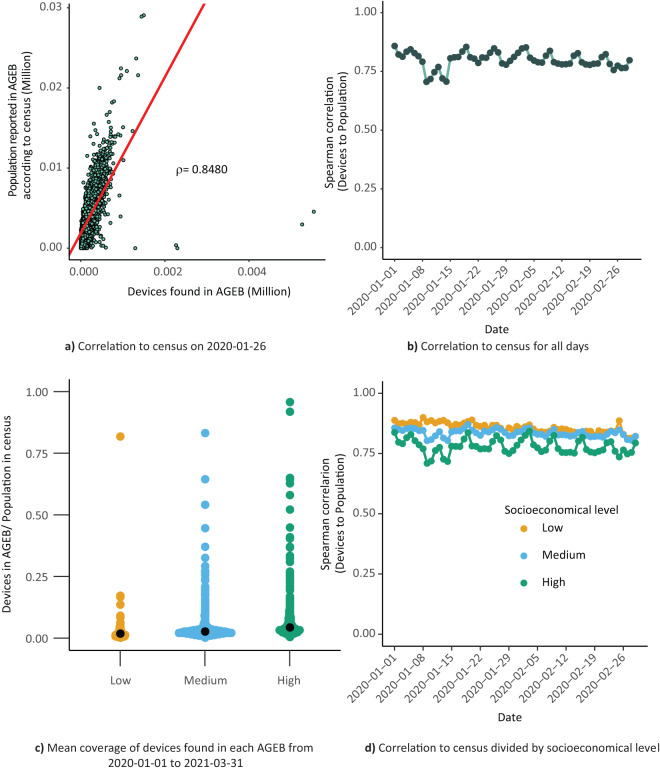
Distribution of devices in the source dataset correlates to population reported in each AGEB. The devices detected on a day were assigned to a home AGEB, contabilized and compared to the population reported for each AGEB on the 2020 National Population and Housing Census^47^. (**a**) The Spearman correlation of this comparison on a pre-pandemic day (2020-01-26) is shown for detail, as well as (**b**) the correlation of these values across the first two months of 2020. (**c**) Distribution across the population in different socioeconomic strata unveils a higher concentration of AGEBs and devices within the medium and high socioeconomic strata. (**d**) The correlation to the population reported by census, but grouped by reported socioeconomical level of AGEBs, reveals an overrepresentation of AGEBs characterized by a high socioeconomic level.

When comparing the number of devices assigned to each AGEB to the population reported on the census for each AGEB, an acceptable correlation was found as shown in Fig. 5.

Correlation was further inspected to asses if there was a bias by other sociodemographic factors. To inspect the representation of various socioeconomic strata, the AGEBs were categorized into three groups based on their designated socioeconomic status calculated from INEGI^51^. Greater Mexico City encompasses 5545 AGEBs, with 509, 3396, and 1640 falling into low, medium, and high socioeconomic strata, respectively. The average population residing in AGEBs classified as low socioeconomic status is approximately 2,300 individuals, while AGEBs categorized as medium and high socioeconomic strata have populations averaging about 4,100 and 3,400 residents, respectively.

The fraction of unique devices assigned to a home AGEB is determined relative to the population of the corresponding AGEB. Upon examination, it becomes evident that AGEBs with high and medium socioeconomic status concentrate a larger number of devices, as depicted in Fig. 5. However, when evaluating the correlation between the number of devices observed in home AGEBs in relation to the census data, an improved correlation is observed in AGEBs falling within the low and medium socioeconomic levels. This enhanced correlation contrasts with a lower correlation observed in AGEBs with high socioeconomic status, possibly attributed to an overrepresentation of devices in such AGEBs, potentially due to individuals in this socioeconomic status possessing more than one device.

### Comparison to reference data

Google generated Community Mobility Reports during the COVID-19 crisis to illustrate changes in population movement trends across different regions of the world. These openly accessible data consist of reports for various countries and provinces, highlighting the relative changes in recorded visits to different categories of places: retail and recreation, grocery and pharmacy, parks, transit stations, workplaces, and residential areas.

For each category, the reference point was the observed dynamics over five weeks, from January 3rd to February 6th. The median value for each day of the week was calculated during this time frame. Subsequently, from February 15th to December 31st, the relative change observed in comparison to the baseline was established.

**Fig. 6:**
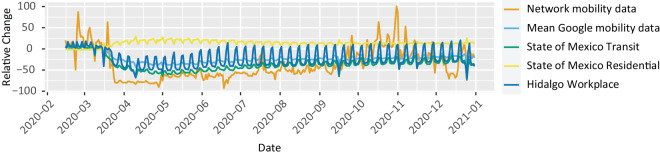
Comparison between the overall mobility observed in mobility networks (the summation of all weights in the network corresponding to each day) and Google’s mobility reports. Google adopts a distinct segmentation approach, quantifying visits to places within specific categories. This figure contrasts our data, featuring three examples of Google’s reports related to Residential points in the State of Mexico, Transit stations in the State of Mexico, and Workplaces in the State of Hidalgo. Additionally, an average of Google’s reports across categories and the states comprising the Greater Mexico City region (Hidalgo, State of Mexico, and Mexico City) is presented.

To compare our data, we replicated Google’s calculation, considering the same time frame as a reference and calculating the daily median within our dataset (as shown in Fig. 6). However, when evaluating our data against Google’s reports, three crucial points must be considered. First, Google’s reports for Mexico are organized by state, not by municipality or AGEBs. This limitation hinders an appropriate comparison for the Greater Mexico City area, which, as detailed earlier, encompasses municipalities from three different states (Mexico City, State of Mexico, State of Hidalgo). Secondly, Google employs particular categories to quantify the difference between the baseline and the analyzed period of 2020; specifically, they calculate the relative change in recorded visits to locations corresponding to retail and recreation, grocery and pharmacy, parks, transit stations, workplaces and residential. In contrast, our study does not identify locations serving as pivot points to measure changes in population dynamics. Lastly, Google’s report documentation recommends caution when contrasting data from different regions, as local differences could lead to inappropriate comparisons.

The change in territorial division and measurement focus prevents a direct comparison of our data with the information in these reports. Figure 6 illustrates three examples contrasting our data with that for states partially forming part of Greater Mexico City. Due to different categories of points of interest, these data records vary significantly among themselves. The three examples correspond to Residential points in the State of Mexico, Transit stations in the State of Mexico, and Workplaces in the State of Hidalgo.

We computed the average of Google’s reports for all categories within the three federal entities that comprise the metropolitan area of Greater Mexico City. This average is also shown in Fig. 6, where we observe a Spearman correlation coefficient of 0.77 with our data. Veraset’s data collection methodology (Fig. 2) could potentially translate into data fluctuations within our networks; in the comparison with Google’s data, a significant change can be spotted from October 2020 onward.

Considering the distinct nature of both datasets in terms of design, our data appears reasonably compatible with Google’s reference data. Furthermore, our dataset surpasses the capabilities of Google’s reports in capturing mobility patterns in terms of granularity, as detailed intra-mobility analyses can be conducted within Greater Mexico City or any user-defined region. In this way, the dataset reported in the present work enables the examination of inter-mobility patterns between regions and facilitates the discernment of changes over short periods, which makes it an invaluable resource for mobility studies.

### Event detection

**Fig. 7:**
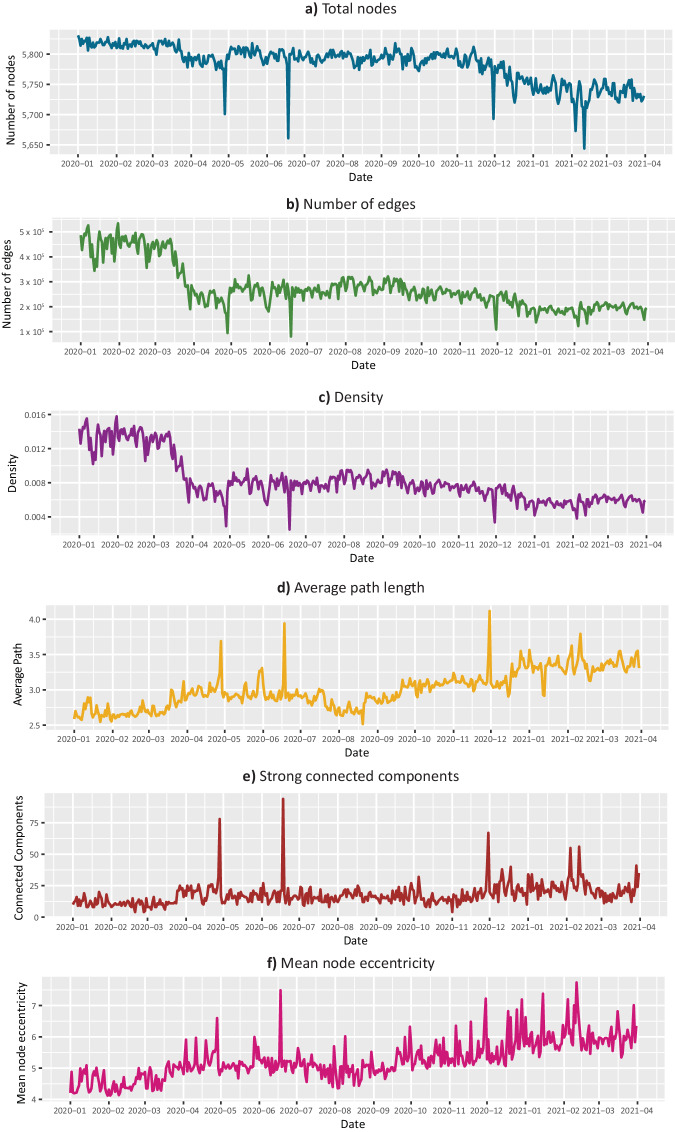
Mobility network’s properties change across time. Basic network statistics were calculated for the networks corresponding to each day between January 2020 and March 2021.

To estimate the quality of the data, a time series analysis of different metrics in the network collection was performed. Metrics considered for this analysis were total node and edge counts, density, average path length, strong connected components and node eccentricity. Figure 7 shows a graphical representation of these metrics for the network collection. These and additional network statistics are available as a table on this publication’s repository^50^. As expected, these metrics fluctuate across time, and further analysis of these changes may help understand the mobility patterns underlying. For example, we decided to build a series that considers the total sum of weights for each day in the collection networks. Then, we used the PersistAd model of the ADTK library in Python, which detects anomalies using a previous time window as a reference; a point is labeled as anomalous if the change in value it represents with respect to the median of the previous period is abnormally large. For our experiment we consider a seven-day window and the option to detect anomalies related to both positive and negative changes.

**Fig. 8:**
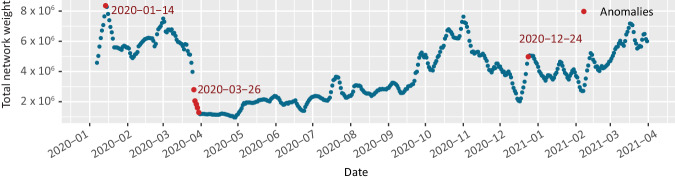
Total sum of edge weights of the networks in each mobility network. This value reflects the general displacement observed in Greater Mexico City on the indicated date. Dates detected as anomalies have been marked in red.

Figure 8 shows the time series obtained and the dates that, according to the selected model represent an anomaly. As can be seen, the algorithm identified January 14, 2020, the period from March 26 to 30, 2020 and December 24th, 2020 as anomalous values.

**Fig. 9:**
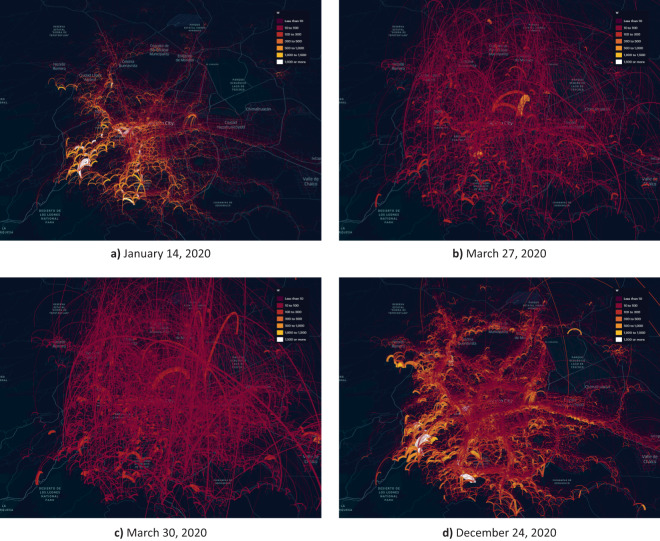
Mobility between zones (AGEBs) of Greater Mexico City on days identified as anomalies during the time period between January 1, 2020 and March 31, 2021. January 14 shows an increase in mobility possibly related to the return to normal activities after the winter vacations. In contrast, the period of March 27–30 shows a clear decrease in population movement, following the initiation of government-recommended security measures due to the pandemic. Movement increases again in December, specially on the occasion of Christmas.

The date identified in January, which corresponds to an increase in mobility, could be related to the return to normal activities (work, school) after the winter vacation period. On the other hand, the algorithm identifies a noticeable decline in mobility during March 2020. The dates identified in this period coincide with the outbreak of COVID-19 cases in the country and with the start of the National Healthy Distance Program (Jornada Nacional de Sana Distancia) implemented by the federal government in Mexico, which officially began on March 23rd^52^. Finally, the algorithm identifies a new increase in mobility in the city at the end of the year; these changes in movement were most likely related to the Christmas holidays. The mobility between the different AGEBs on the identified dates is shown in Fig. 9.

Beyond the specific dates pinpointed by the algorithm, a significant reduction in overall mobility within the region is noticeable starting from March 2020. This example serves as an intuitive validation of the data quality underpinning the networks and the network construction process. It appears that the data effectively capture significant shifts in population mobility that occurred in Mexico following the health contingency.

Furthermore, this example may also prompt questions that could be explored through in-depth analysis of the dataset. Utilizing more advanced tools for complex network analysis or cross-referencing these data with other sources of AGEB-related information could unveil more intricate patterns. Such an approach would allow us to contrast the disruption that daily life experienced in different sectors of the city, providing valuable insights into the varying impacts of the pandemic.

It is important to underscore that our data, much like other datasets of a similar nature, are subject to limitations imposed by the data collection and sampling strategy employed by the data provider. Nevertheless, based on our exploration, the dataset can prove valuable for analyzing population mobility within the Greater Mexico City metropolitan area.

## Usage Notes

The dataset is available in CSV format, making it easily accessible without the need for specialized software. While importing the data, it is important to note that the networks are directed and weighted.

To enhance the richness of the information presented within the networks, we recommend utilizing databases provided by the Mexican National Institute of Statistics, Geography and Informatics (Instituto Nacional de Estadística, Geografía e Informática de México - INEGI). The definition of localities within our dataset aligns with the division proposed by INEGI, ensuring compatibility with results from various tools and resources available on their website^39,53^.

## Data Availability

DuckDB and Python were employed for constructing the mobility networks. The entire network dataset, along with the code utilized for network construction and supplementary tables can be found at: 10.17605/OSF.IO/GWQ6U^50^.
