# Author Correction: Mobility networks in Greater Mexico City

**DOI:** 10.1038/s41597-024-03630-4

**Published:** 2024-07-15

**Authors:** Marisol Flores-Garrido, Guillermo de Anda-Jáuregui, Plinio Guzmán, Amilcar Meneses-Viveros, Alfredo Hernández-Álvarez, Erika Cruz-Bonilla, Maribel Hernández-Rosales

**Affiliations:** 1 Escuela Nacional de Estudios Superiores Unidad Morelia, Universidad Nacional Autónoma de México, Antigua Carretera a Pátzcuaro 8701, Indeco la Huerta, Ciudad de México, 58190 Michoacan Mexico; 2 National Institute of Genomics Medicine, Periferico Sur 4809, Arenal Tepepan, Tlalpan, 14610 Mexico City, Mexico; 3 National Council for Science and Technology, Av. Insurgentes Sur 1582, Colonia Crédito Constructor, Benito Juárez, Mexico City, Mexico; 4 Estornuda.me, Mexico City, Mexico; 5 Center for Research and Advanced Studies of IPN, Av Instituto Politécnico Nacional 2508, San Pedro Zacatenco, Gustavo A. Madero, 07360 Mexico City, Mexico; 6 Center for Genomics Sciences, Universidad Nacional Autónoma de México, Avenida Universidad s/n, Universidad Autonoma del Estado de Morelos, 62210 Cuernavaca, Morelos Mexico; 7 Center for Research and Advanced Studies of IPN, Irapuato Unit, Libramiento Norte Carretera Irapuato León Kilómetro 9.6, 36821 Irapuato, Guanajuato Mexico

**Keywords:** Scientific data, Computer science

Correction to: *Scientific Data* 10.1038/s41597-023-02880-y, published online 18 January 2024

In this article the captions to Figures 4 and 5 were inadvertently swapped. The original article has been corrected.

